# Supplementary material for: Splice-Junction-Based Mapping of Alternative Isoforms in the Human Proteome
Source: Cell Rep. Author manuscript; Available in PMC 2020 Jan 15. (PMC6961840; doi:10.1016/j.celrep.2019.11.026)
Supplement: 2 [file NIHMS1546469-supplement-2.zip › DF1/PXD006675/MitralValve/MitralValve_6_DPP7_FASPHSPQGPGPR.pdf]

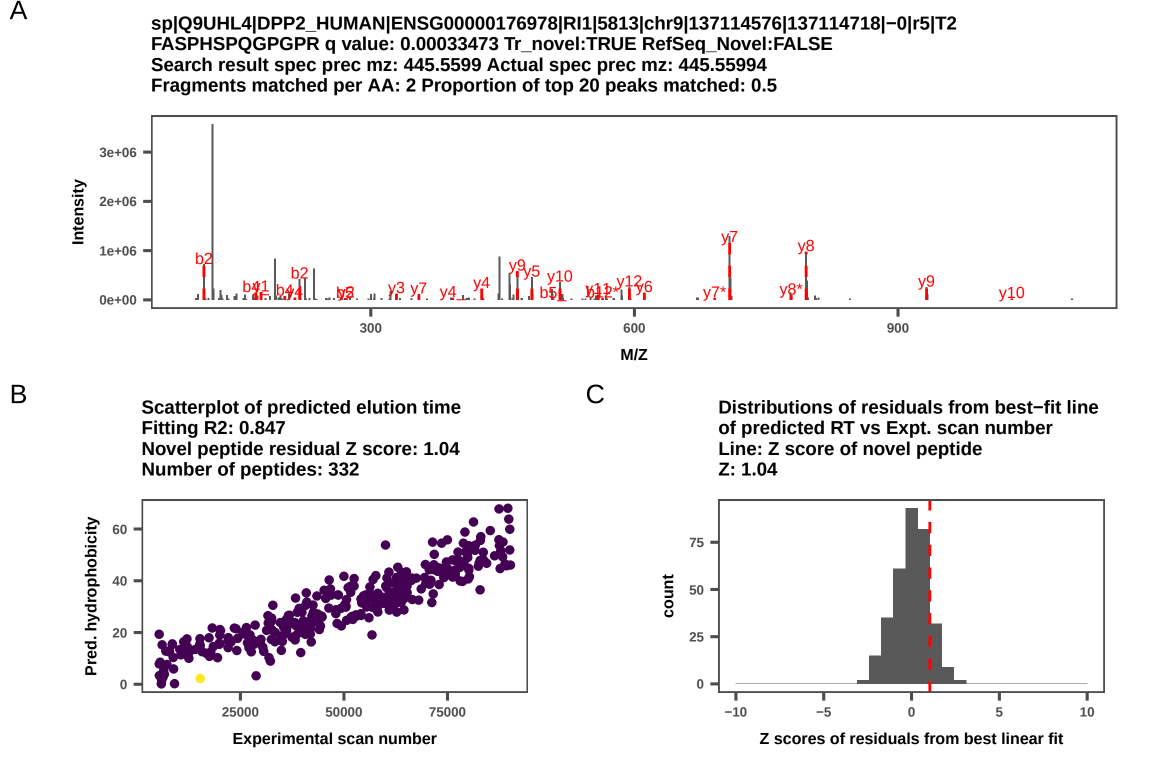
A
sp|Q9UHL4|DPP2_HUMAN|ENSG00000176978|RI1|5813|chr9|137114576|137114718|−0|r5|T2
FASPHSPQGPGPR q value: 0.00033473 Tr_novel:TRUE RefSeq_Novel:FALSE
Search result spec prec mz: 445.5599 Actual spec prec mz: 445.55994
Fragments matched per AA: 2 Proportion of top 20 peaks matched: 0.5
Intensity
3e+06
2e+06
1e+06
0e+00
b2
b2
y3
y7
y4
y4
y9
y5
y10
b5
y12
y6
y7*
y7
y8*
y8
y9
y10
300
600
900
M/Z
B
Scatterplot of predicted elution time
Fitting R2: 0.847
Novel peptide residual Z score: 1.04
Number of peptides: 332
Pred. hydrophobicity
60
40
20
0
25000
50000
75000
Experimental scan number
C
Distributions of residuals from best−fit line
of predicted RT vs Expt. scan number
Line: Z score of novel peptide
Z: 1.04
count
75
50
25
0
−10
−5
0
5
10
Z scores of residuals from best linear fit
